# PFTuner: An Efficient and Effective Multi-Objective Configuration Tuning Framework Adaptive to Different Software Systems

**DOI:** 10.3390/s26103028

**Published:** 2026-05-11

**Authors:** Jie Feng, Mingdong He, Lei Jin, Hui Dou

**Affiliations:** 1 Guangdong Power Grid Co., Ltd., Guangzhou 510699, China; fengjie@gd.csg.cn (J.F.); 13825004075@139.com (M.H.); 2 School of Computer Science and Technology, Anhui University, Hefei 230601, China; jinlei@stu.ahu.edu.cn

**Keywords:** configurable software systems, parameters tuning, multi-objective optimization, Bayesian Optimization

## Abstract

Software systems often expose a large number of configurable parameters to satisfy diverse application requirements and deployment scenarios. Given the intricate dependencies between parameters, manually finding a well-performing configuration is a daunting task even for experienced operators. Most existing automatic tuning approaches treat the problem as a single-objective search, leaving critical concerns such as energy consumption and reliability as afterthoughts. Although recent studies have explored multi-objective configuration tuning, they still face several challenges, including handling conflicting objectives, balancing search effectiveness and efficiency, and adapting to heterogeneous configuration spaces across different software systems. To address these issues, we propose PFTuner, an efficient and effective multi-objective configuration tuning framework adaptive to diverse software systems. PFTuner consists of three collaborative modules, namely Configuration Generator, Configuration Evaluator, and Sample Collector, which operate iteratively to continuously improve configuration quality. In particular, we design a novel multi-objective optimization algorithm that effectively models heterogeneous configuration spaces and improves the balance between optimization quality and search efficiency. We evaluate PFTuner on eight software-workload scenarios deployed on a local cluster and compare it with several representative state-of-the-art baselines. Experimental results show that PFTuner consistently achieves higher-quality Pareto fronts and better search efficiency, while also demonstrating strong adaptability across different software systems and workloads.

## 1. Introduction

Over the past decade, the explosive growth of web services, e-commerce, and connected devices has pushed organizations to rethink how they store and process data at scale, making platforms like Spark, HBase, and MySQL gradually become mainstream development trends. Numerous enterprises have begun deploying large-scale software systems, such as Spark, HBase, and MySQL, to enhance their data processing capabilities and ensure stable business operations. These systems are typically regarded as configurable software systems, which expose a large number of tunable parameters during the design phase to support diverse runtime objectives. To meet diverse user demands, software developers introduce numerous configurable parameters during the design phase, aiming to help users achieve predefined functional or non-functional goals [1]. Taking system performance as an example, a bad configuration may lead to system performance degradation, which in turn affects service quality and can lead to significant economic losses for enterprises. However, given the inherent complexity of software systems, parameter configuration typically requires users to possess a profound understanding of the system’s mechanisms. Therefore, it is necessary for organizations to find the best configuration without human intervention, and the research community has responded with growing interest in automatic parameter tuning over the past several years.

Traditional software configuration optimization efforts [2,3,4,5,6,7] have largely focused on computational performance tuning, prioritizing performance improvements while overlooking other functional attributes of the system, such as energy consumption, security, and reliability. However, with the continuous development and refinement of software systems and a shift in application philosophies, user requirements for various software operational attributes have become more balanced. For instance, the focus on green computing has made energy consumption a key optimization dimension. Persisting in enhancing only a single operational attribute of a software system can easily lead to performance surplus while causing unnecessary energy waste. In addition, single-objective configuration tuning not only increases operational costs for enterprises but may also pose potential threats to the overall service quality of the software system. As introduced in [8], a user might change a specific parameter in MySQL to achieve better performance, but this could come at the risk of losing transactions in the event of a system crash. Consequently, to address these more balanced and diverse optimization needs, there is an urgent need for the joint automatic tuning of multiple functional attributes of software systems during runtime.

To achieve multi-objective optimization, researchers have attempted various solutions. Some works convert the problem into a single-objective one by randomly assigning weights to each optimization objective [9,10]. However, pinning down a sensible weight vector ahead of time is harder than it sounds, since the right balance depends on the very Pareto front that has not yet been discovered. Other works have tried to adapt traditional multi-objective algorithms (such as NSGA-II) to configuration optimization scenarios [11,12]. Nevertheless, these methods also face severe challenges.

CH1: How to effectively balance conflicting optimization objectives? Software tuning often involves competing goals, such as maximizing throughput while minimizing energy consumption. These objectives typically exhibit complex, non-linear relationships that cannot be easily resolved by simple linear weighting. Traditional single-objective methods fail to capture these trade-offs, often optimizing one metric at the expense of others. Designing a mechanism to decouple and model these conflicting objectives simultaneously is the primary challenge.

CH2: How to find the best balance between search efficiency and effectiveness? Traditional multi-objective optimization methods (such as multi-objective evolutionary algorithms, or MOEAs) typically generate a random initial population at startup. When faced with complex configuration parameter spaces, this often results in many invalid configurations. Furthermore, the computational paradigm of these algorithms inherently involves non-negligible computational time overhead. More importantly, providing the algorithm with sufficient sample points necessitates the evaluation of a large number of configurations. For software systems, the time cost associated with repeatedly running benchmarking programs is often unacceptable, making it difficult for the algorithm to explore effective configurations within the constrained time.

CH3: How to ensure cross-system adaptability with the heterogeneous configuration spaces? To solve the aforementioned efficiency problem, researchers have proposed optimization methods based on heuristic strategies. Although these methods can guide exploration and achieve good results in specific systems (like Spark) using these heuristics, their optimization effectiveness is often limited when dealing with the heterogeneous configuration spaces of different types of software systems. This is particularly true in software systems with unevenly distributed configuration spaces (such as MySQL and HBase), where the guiding capability of heuristic strategies is significantly weakened. This results in poor adaptability, limiting the effectiveness of optimization, and potentially even causing the method to degenerate into a single-objective optimization approach.

To address the aforementioned challenges, this paper proposes PFTuner, an efficient and effective multi-objective configuration tuning framework with adaptability to different software systems. PFTuner primarily consists of three modules: Configuration Generator, Configuration Evaluator, and Sample Collector. These modules cooperate with each other and work in an iterative manner. Specifically, we propose a novel multi-objective optimization algorithm with adaptability to heterogeneous configuration spaces in the Configuration Generator module. The core insight of this algorithm is "Decouple and Guide". It adopts a pseudo-agent cooperative strategy to decouple conflicting objectives (e.g., performance vs. energy) into separate surrogate models, allowing flexible modeling of complex relationships. More importantly, to address the poor adaptability of heuristic strategies in heterogeneous spaces, it introduces a quantitative guidance mechanism based on Pareto front quality. Instead of blindly accepting agents’ candidates, it employs a system-agnostic metric MHD (Modified HyperVolume-based Difference) [13] to rigorously calculate the potential "improvement degree" of each candidate configuration. By dynamically selecting the configuration that maximizes the theoretical expansion of the Pareto front, it can ensure that every expensive evaluation contributes significantly to the optimization goal, thereby achieving robust adaptability across diverse software systems. Detailed description of the proposed algorithm as well as the tuning process of PFTuner can be found in Section 4.

To validate the effectiveness and efficiency of PFTuner, we conducted comprehensive experiments on three widely used software systems: Spark, MySQL, and HBase. The experimental results demonstrate that PFTuner significantly outperforms the representative baseline methods in both search efficiency and Pareto-front quality. For instance, for the Spark-PageRank workload, compared with the best-performing baseline method, PFTuner can achieve 10.59% less execution time and even slightly lower energy consumption. It is also able to consume 53.04% less energy with a close execution time. Notably, the comprehensive average optimization time overhead per iteration over three Spark workloads of PFTuner is reduced by as much as 194.14 s compared with the baselines. In addition, PFTuner also exhibits robust adaptability across a total of eight different software-workload scenarios with heterogeneous configuration spaces. The contributions of this paper are summarized as follows:

- We propose a novel multi-objective optimization algorithm for configuration generation. To address the aforementioned challenges, it utilizes a pseudo-agent cooperative strategy to resolve the conflicts between optimization objectives and incorporates the MHD metric to rigorously quantify the contribution of each candidate configuration to guide the search direction.
- Based on the proposed multi-objective algorithm, we design and implement PFTuner, an efficient and effective multi-objective configuration tuning framework adaptive to different software systems.
- We conduct extensive experiments with a total of eight different software-workload scenarios. The results demonstrate that PFTuner outperforms the representative baseline methods in finding high-quality Pareto fronts as well as search efficiency. In addition, it also exhibits superior adaptability across diverse software systems.

The remainder of this paper is organized as follows: Section 2 introduces the related work. Section 3 introduces the preliminaries and details the problem formulation. After that, Section 4 describes the design and implementation of PFTuner. Section 5 describes the experimental setup, and Section 6 presents the experimental results and analysis. Finally, Section 7 concludes this paper.

## 2. Related Work

Model-based Optimizer. In Model-based methods, the optimizer typically starts by collecting sample data offline, followed by constructing a predictive model using machine learning algorithms. Subsequently, search algorithms are applied to explore the configuration space based on the predictive model to identify and determine the optimal configuration [2,3,4,5,14]. To reduce the required size of the training dataset, researchers often preselect a subset of critical configuration parameters to shrink the configuration space [15,16]. Ref. [17] uses performance data with a CART-based random forest to identify key database configuration parameters and capture their nonlinear interactions. Unfortunately, due to the dynamic variation in workload types and input dataset sizes in real-world environments, model-based approaches inevitably face frequent model retraining, which severely limits their practical applicability in software system tuning.

Search-based Optimizer. Unlike model-based methods, search-based methods treat the optimization objective as a black-box function and explore the configuration space online following specific search strategies [6,7]. Some studies [18,19] have leveraged deep reinforcement learning (DRL) to navigate the high-dimensional configuration spaces of cloud databases. Trummer [20] proposes an automated cloud database tuning tool that uses NLP to extract knowledge from documentation and reinforcement learning to guide configuration optimization. However, the effectiveness of DRL largely depends on the quality of the experience pool, which usually requires substantial training data.

Compared with DRL-based methods, Bayesian Optimization (BO) relies less on surrogate model accuracy and achieves near-optimal configurations with significantly lower sampling cost. Consequently, BO has been widely adopted for software system configuration tuning. For example, ref. [21] built an ensemble of BO models with diverse hyperparameters to explore the configuration space, while ref. [22] developed CGPTuner, a BO-based tool achieving strong performance on Cassandra and MongoDB. Ref. [23] designed and implemented OnlineTune, a tool aimed at safely tuning configuration parameters of cloud databases in cloud environments. The key idea is to explicitly incorporate environmental factors as contextual features and apply contextual Bayesian Optimization to explore the configuration space, thereby enabling precise and efficient configuration adjustment. In this paper, the proposed multi-objective optimization algorithm can also be treated as a BO-based solution target for efficient and effective multi-objective configuration tuning adaptive to different software systems.

Multi-objective Optimizer. While the aforementioned model-based and search-based approaches have achieved significant success in optimizing single performance metrics (e.g., latency or throughput), they often overlook the critical trade-offs, such as balancing performance with energy consumption; merely maximizing a single attribute may lead to resource over-provisioning or stability risks, necessitating the adoption of multi-objective optimization (MOO) strategies.

To address conflicting objectives, early works attempted to simplify the problem by converting it into a single-objective one via scalarization. For instance, ref. [9] proposed Flash, a sequential model-based optimization strategy that attempts to cover the Pareto front by randomly assigning weights to different objectives in each iteration. Similarly, ref. [10] introduced Karasu, a collaborative optimization method designed for efficiently configuring resources in large-scale data analytics clusters. When handling multi-objective optimization problems, Karasu constructs an individual Gaussian Process model for each optimization objective and constraint. It then integrates these single-objective models using a weighted-ranking Gaussian Process ensemble, through which it recommends the optimal configuration for a given workload. However, as noted in recent studies, determining appropriate weight assignments in advance is practically difficult and often fails to capture the complex, non-linear Pareto frontiers of real-world software systems.

To directly approximate the Pareto set, researchers have applied multi-objective evolutionary algorithms (MOEAs). For instance, ref. [12] proposes a multi-objective framework for tuning configurations under concurrent workloads, using a stacked neural network for performance modeling and NSGA-II for optimization. Ref. [24] integrates a decomposition-based multi-objective evolutionary algorithm with an AdaBoost-based performance model to automatically explore Spark’s cloud configuration space. While effective in exploring large spaces, these population-based methods typically require a vast number of function evaluations to converge. In the context of software tuning, where each evaluation (e.g., running a Spark benchmark) is computationally expensive, the time cost of standard MOEAs is often prohibitive. To improve sample efficiency, recent advancements have introduced multi-objective Bayesian Optimization (MOBO) into software tuning. For instance, ref. [11] proposed a Gauss–Pareto-based multi-objective optimization approach, which employs a Kriging model to balance execution time and memory usage. Ref. [25] developed an SLA-aware microservice framework that balances service-level objectives and resource cost. Their method applies multi-objective Bayesian Optimization to explore the high-dimensional configuration space and identify Pareto-optimal configurations that effectively meet both performance and cost goals.

However, a critical limitation shared by these MOBO methods is their reliance on acquisition functions or heuristic selection criteria that implicitly assume a degree of regularity in the configuration space. When faced with the heterogeneous and unevenly distributed spaces characteristic of systems like MySQL and HBase (where performance-critical parameters are clustered in sparse, irregular regions while others have negligible effect), standard acquisition functions lose their ability to distinguish promising regions from uninformative ones. The result is that methods performing well on Spark, whose parameter space is relatively uniform, often degrade significantly when transferred to other systems. PFTuner addresses this limitation through the coordinated design of its core components. First, the pseudo-agent cooperative strategy assigns an independent Gaussian Process surrogate model to each optimization objective, allowing the system to capture the distinct landscape of each metric separately and propose candidates that represent diverse trade-offs rather than collapsing multiple objectives into a single model that may obscure their individual structure. Second, rather than relying on acquisition heuristics tied to the geometry of the parameter space, PFTuner employs the Modified HyperVolume-based Difference (MHD) metric [13] to evaluate each candidate purely in terms of its geometric contribution to the Pareto front in the objective space. Because MHD quantifies improvement based solely on the shape and distribution of the current solution set, it remains an effective guide regardless of how parameters are distributed across different software systems. Finally, a threshold filtering mechanism ensures that only candidates with meaningful MHD improvement scores trigger real benchmark evaluations, preventing the limited budget from being consumed by marginal explorations. Together, these three components allow PFTuner to navigate configuration spaces as structurally different as those of Spark, MySQL, and HBase without requiring any system-specific adaptation.

## 3. Preliminaries

### 3.1. Configurable Software Systems and Their Characteristics

Modern software platforms such as big data analytics engines, relational databases, and NoSQL stores are built around a common design philosophy: exposing a large set of tunable parameters so that operators can adapt system behavior to their specific workloads and deployment environments [1]. We refer to such platforms as configurable software systems. In this work, we focus on three representative systems that span distinct software categories: Apache Spark 2.2.2 [26], a widely deployed in-memory computing framework for large-scale data analytics; MySQL 5.7.32 [27], a relational database management system central to transactional workloads; and Apache HBase 2.0.5 [28], a distributed NoSQL store optimized for sparse, wide-table access patterns. Despite serving different purposes, all three share a set of characteristics that make their configuration spaces particularly challenging to optimize: (1) high-dimensional and heterogeneous parameter spaces; (2) black-box objective functions; and (3) conflicting tuning objectives. Taken together, these characteristics define the core difficulty of the problem addressed in this paper: searching for a high-quality approximation of the Pareto-optimal configuration set within a strictly limited evaluation budget, across systems whose configuration spaces are structurally different from one another.

### 3.2. Bayesian Optimization

Bayesian Optimization [29] is a machine learning-based, black-box optimization algorithm suitable for optimization problems where the objective function’s expression is unknown, non-differentiable, or computationally expensive to evaluate. The algorithm operates by constructing a probabilistic surrogate model (typically a Gaussian Process) to predict the function’s value and its associated uncertainty at arbitrary points. It then utilizes these predictions to define an Acquisition Function. The acquisition function is used to evaluate the potential value of each sampling point, and its extreme point indicates the most promising location for the next sample. By balancing the trade-off between “Exploitation” (searching near known optima) and “Exploration” (probing regions of high uncertainty), Bayesian Optimization can approximate the optimal solution of the objective function with a relatively small sampling overhead.

Gaussian Processes (GPs) are a core component of Bayesian Optimization. A GP is a stochastic process defined by its mean function and covariance function (kernel function) [30]. A key property of Gaussian Processes is that if the prior distribution of the black-box function is assumed to follow a Gaussian Process, then the posterior distribution of the function at a finite number of sampled points will also be a Gaussian Process. As more sample points are evaluated, the variance of the posterior Gaussian Process gradually decreases, and the estimated mean increasingly approaches the target black-box function being approximated.

### 3.3. Multi-Objective Optimization

A continuous, unconstrained multi-objective optimization problem (considering minimization without loss of generality) can be formulated as

(1) $${\mathrm{Min}}_{x\in \Omega }F\left(x\right)={\left(f_{1}\left(x\right),f_{2}\left(x\right),\ldots ,f_{m}\left(x\right)\right)}^{T}$$

where x denotes an *n*-dimensional decision vector within the decision space Ω, and *m* represents the number of objective functions. The mapping $F:\Omega \to R^{m}$ projects the decision space to the objective space.

In practice, these objective functions are often conflicting, meaning that improving one objective typically leads to the degradation of another. As a result, multi-objective optimization does not aim to find a single optimal solution, but rather a set of trade-off solutions that reflect different balances among objectives. This concept is formally captured by Pareto optimality.

Specifically, given two decision vectors x and y, x is said to dominate y if and only if x is no worse than y in all objectives and strictly better in at least one objective, i.e.,

$$\left(\forall i\in \left\{1,\ldots ,m\right\}:f_{i}\left(x\right)\leq f_{i}\left(y\right)\right)\wedge \left(\exists j\in \left\{1,\ldots ,m\right\}:f_{j}\left(x\right)<f_{j}\left(y\right)\right).$$

A solution x is considered Pareto optimal if there exists no other solution in Ω that dominates it. The collection of all Pareto optimal solutions forms the Pareto-optimal set, denoted as PS. Correspondingly, the image of this set in the objective space constitutes the Pareto-optimal front, defined as PF={F(x)∣x∈PS}. The Pareto front provides a comprehensive characterization of the trade-offs among objectives, and serves as the primary target for most multi-objective optimization algorithms.

### 3.4. Problem Statement

This research targets the black-box, expensive-to-evaluate, and heterogeneous multi-objective optimization problem for configurable software systems under a strictly limited evaluation budget. For the space of the screened *D*-dimensional configuration parameter, let $x_{d}$ denote the *d*-th configuration parameter (d=1,2,…,D), where the parameter $x_{d}$ takes a value from a finite domain $Dom(x_{d})$. In practical systems, $x_{d}$ can be a variable with a real value, or a categorical or enumerated variable. For non-real-valued variables, this section introduces index values to represent the corresponding categories and sets this index as the parameter’s value to facilitate numerical calculations in the subsequent optimization. Therefore, the complete candidate configuration space can be defined as follows.

(2) $$X=\prod_{d=1}^{D}Dom\left(x_{d}\right)$$

Assuming that the user is concerned with *M* different system operational attributes and $f_{m}$ represents the *m*-th objective attribute to be optimized, the corresponding attribute metric recorded after running each configuration x∈X in the candidate space is $f_{m}\left(x\right)$. Thus, the joint multi-attribute objective optimization problem for configurable software systems (taking minimization as an example) is formalized as

(3) $$\begin{array}{c} PS=argmin_{x\in X}F\left(x\right)\\ F\left(x\right)=\left\{f_{1}\left(x\right),f_{2}\left(x\right),\ldots ,f_{m}\left(x\right),\ldots ,f_{M}\left(x\right)\right\}\\ s.t.\;evaluation\;times\leq ET_{\max} \end{array}$$

Given the high cost of evaluating each configuration in a real environment, the time spent on the optimization process must be limited, which means that the number of observed samples should be less than the maximum evaluation budget $ET_{\max}$. The ultimate goal is to efficiently explore the candidate configuration space to identify a high-quality approximation of the Pareto-optimal set (PS) within a strictly limited evaluation budget.

## 4. The PFTuner Framework

### 4.1. Overview of PFTuner

**Figure 1:**
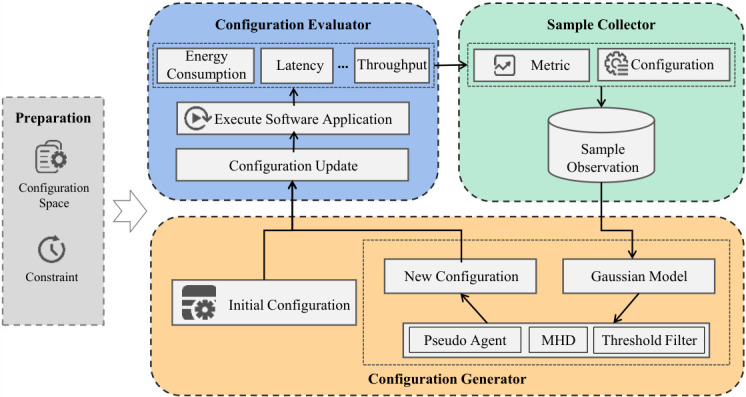
Overview of the proposed multi-objective configuration tuning framework PFTuner.

PFTuner is an online, iterative search framework designed specifically for the multi-objective configuration tuning problem in software systems, where three domain-specific characteristics—high evaluation cost, strict budget constraints, and heterogeneous configuration spaces—distinguish it from general-purpose multi-objective optimization settings. Unlike general-purpose optimizers such as NSGA-II or USeMO, which are designed to be broadly applicable without assuming a specific evaluation context, each component of PFTuner is explicitly motivated by practical challenges in software tuning. The framework aims to identify a high-quality Pareto-optimal set under a limited evaluation budget. As illustrated in Figure 1, PFTuner follows an iterative optimization loop composed of three tightly coupled modules.

At the core of the framework is the Configuration Generator, which is responsible for recommending promising configuration candidates for evaluation based on the currently observed samples. This module implements the proposed multi-objective optimization algorithm, which is guided by improvements in Pareto front quality. To effectively balance conflicting objectives, it adopts a pseudo-agent cooperative strategy, allowing different optimization targets to be explored in a coordinated manner. In addition, the Modified HyperVolume-based Difference (MHD) [13] metric is introduced to quantitatively evaluate the contribution of candidate configurations and guide the search toward regions with higher potential improvements.

Once a new configuration is generated, it is passed to the Configuration Evaluator, which serves as the interface between the optimization algorithm and the target software system. This module applies the configuration to the system and executes the specified benchmark workloads to obtain runtime performance metrics, such as execution time, energy consumption, and throughput. Considering the inherent noise in system measurements, each configuration is evaluated multiple times, and the aggregated results are used to improve robustness and reduce variance in observations.

The resulting performance metrics, together with their corresponding configurations, are then handled by the Sample Collector. This module is responsible for maintaining the observation dataset by persistently storing each 〈configuration, metric set〉 pair. After incorporating new samples, the surrogate models associated with each optimization objective are updated accordingly. The updated models are then used by the Configuration Generator in the next iteration, forming a closed optimization loop.

Through the interaction of these three modules, PFTuner continuously refines its understanding of the configuration space and progressively approaches the Pareto-optimal front within the given budget constraints.

### 4.2. The Proposed Multi-Objective Optimization Algorithm in the Configuration Generator Module of PFTuner

To effectively explore the configuration space with a limited evaluation budget, we propose a novel multi-objective optimization algorithm in PFTuner. Specifically, it builds upon a pseudo-agent cooperative strategy to handle objective conflicts, introduces an innovative MHD-based guidance mechanism to steer the search direction efficiently, and incorporates a threshold filtering mechanism to efficiently prune marginal candidates.

#### 4.2.1. The Pseudo-Agent Cooperative Strategy

To resolve the conflict between optimization objectives, we propose the pseudo-agent cooperative strategy. While maintaining independent surrogate models for individual objectives is not new in the MOBO literature, the way these models interact and collectively drive the search in PFTuner differs fundamentally from standard practices. In standard MOBO methods, per-objective surrogate models ultimately serve a single unified acquisition function, which can cause certain objectives to dominate the search direction when the configuration space is heterogeneous or the Pareto front is irregularly shaped. In PFTuner, by contrast, each pseudo-agent independently maximizes its own acquisition function and proposes the candidate it finds most promising from its own single-objective perspective. The proposals from all *m* agents are pooled to form the candidate set $X_{c}$, which by construction spans diverse trade-off regions of the objective space without any single objective dominating the nomination process. The subsequent MHD-based selection step then identifies which nominated candidate contributes most to expanding the Pareto front. In fact, it is this closed-loop "nominate-then-select" design rather than the use of independent surrogates per se that constitutes the distinguishing contribution of the pseudo-agent strategy and proves particularly effective when the underlying configuration space is heterogeneous.

Each agent maintains its surrogate model as a Gaussian Process (GP) [30]. This choice is deliberate rather than incidental, and is motivated by the specific constraints of the software configuration tuning problem. Unlike deterministic models such as random forests or neural networks, a GP provides not only a predicted mean $\mu_{i}\left(x\right)$ but also a calibrated uncertainty estimate $\sigma_{i}^{2}\left(x\right)$ at any unobserved configuration. This dual output is essential for constructing principled acquisition functions that balance exploitation and exploration—a property that becomes especially valuable when each real-world evaluation requires running a full benchmark workload and may take tens of minutes to complete. Furthermore, compared to deep surrogate models, GPs generalize more reliably under small sample sizes; given that the total evaluation budget in this work is capped at 50, data efficiency is a primary concern. The smoothness and interpretability of GP predictions also make the downstream MHD improvement calculation more stable, as the predicted objective values fed into Equation (4) are less prone to erratic fluctuations than those produced by highly expressive but data-hungry models. It is worth noting that the pseudo-agent framework is not inherently tied to Gaussian Processes. In principle, any surrogate model capable of producing uncertainty-aware predictions, such as Bayesian neural networks or deep kernel learning models, could be substituted into the same agent structure. We leave the exploration of alternative surrogate models as a direction for future work.

Formally, let $A=\{A_{1},A_{2},\ldots ,A_{m}\}$ be the set of pseudo-agents, where each agent $A_{i}$ is explicitly responsible for optimizing the *i*-th objective function $f_{i}\left(x\right)$, such as minimizing execution time or energy consumption. Each agent $A_{i}$ maintains an independent GP surrogate model that approximates the mapping between configurations and its designated objective. Given the current observation dataset *D*, the GP yields both a predicted mean $\mu_{i}\left(x\right)$ and a variance $\sigma_{i}^{2}\left(x\right)$ for any unobserved configuration x, capturing both the expected performance and the remaining uncertainty. In each iteration *t*, every agent independently maximizes its own acquisition function (Expected Improvement (EI) in this work) to identify the configuration most promising from its individual perspective. The proposals from all *m* agents are then pooled to form the candidate set $X_{c}=\left\{x_{c1},x_{c2},\ldots ,x_{cm}\right\}$, which is passed to the MHD-based guidance mechanism for final selection.

#### 4.2.2. MHD-Based Guidance Mechanism

To overcome the limitations of heuristic strategies when faced with heterogeneous configuration spaces, PFTuner adopts a more universal metric-based quantification approach to guide the search. Its core idea is to quantitatively evaluate the “contribution” or “improvement” that each candidate configuration offers to the currently discovered Pareto Front (PF) during the optimization process.

**Figure 2:**
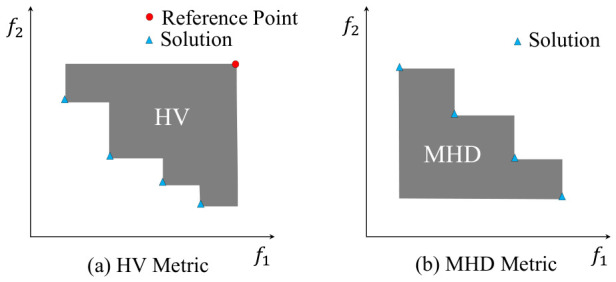
HyperVolume (HV) and Modified HyperVolume-based Difference (MHD) metrics under a two-dimensional objective space.

To evaluate the quality of the obtained Pareto front, it is necessary to introduce corresponding evaluation metrics. HyperVolume (HV) [31] is a widely used metric that measures the volume of the space enclosed by the non-dominated solution set obtained by the algorithm and a predefined reference point. A larger HV value typically signifies that the solution set has better convergence and diversity. However, the HV metric is highly sensitive to the choice of the reference point, and an improper setting can lead to misleading evaluation results. To eliminate this effect, this paper adopts the Modified HyperVolume-based Difference metric [13]. Compared to the traditional HV metric, MHD offers a significant advantage in black-box software tuning: it does not rely on a predefined reference point. As shown in Figure 2, MHD evaluates the quality of a solution set based solely on the shape and distribution of the Pareto front itself, avoiding biases caused by improper reference point settings.

The PFTuner evaluates each candidate $x\in X_{c}$ by calculating its degree of improvement, I(x). This metric represents the marginal gain in the quality of the Pareto front if the predicted values of *x* were added to the current solution set. The improvement function is formally defined as

(4) $$I\left(x\right)=\frac{\left|MHD\left(PF_{t}\right)-MHD\left(PF_{t}^{\prime }\right)\right|}{MHD\left(PF_{t}\right)}$$

Here, ${\mathrm{PF}}_{t}$ represents the existing Pareto front in the *t*-th iteration, and ${\mathrm{PF}}_{t}^{\prime }$ represents the new Pareto front formed after adding the predicted objective values of the candidate configuration x. MHD(·) is the function for calculating the MHD metric value of the corresponding front. In this way, PFTuner does not need to rely on the internal mechanisms of a specific software system or the specific properties of its configuration parameters. Instead, it guides the optimization process based on the universally applicable criterion of Pareto front quality improvement.

#### 4.2.3. Efficiency Optimization via Threshold Filtering

Given the “expensive-to-evaluate” nature of software benchmarks defined in Section 3.4, avoiding low-value evaluations is critical for improving overall tuning efficiency. Inspired by the observation that “rare but significant improvements contribute more than frequent but negligible ones” [32], we incorporate a threshold-based filtering mechanism to selectively control evaluation cost.

Specifically, we introduce a minimum improvement threshold τ to regulate whether a candidate configuration should be evaluated. In each iteration, after identifying the most promising candidate $x_{best}$ along with its estimated improvement score $I(x_{best})$, the system makes a decision based on its potential contribution. When $I(x_{best})$ exceeds the threshold τ, the candidate is regarded as sufficiently promising, and a real-world benchmark evaluation is triggered. The resulting observation is then incorporated into the dataset D, and all surrogate models (or agents) are updated accordingly.

In contrast, when $I(x_{best})\leq \tau$, the candidate is considered to provide only marginal improvement. In such cases, the system deliberately skips the expensive evaluation step, thereby reserving the limited evaluation budget for configurations with higher expected impact. This selective evaluation strategy effectively reduces redundant or low-gain explorations without compromising the overall search quality.

In this study, the threshold is empirically set to τ=0.005. This mechanism serves as a lightweight yet effective gatekeeper, enabling PFTuner to prioritize high-impact configurations and improve sample efficiency under strict budget constraints.

### 4.3. Configuration Tuning Process of PFTuner

The complete optimization process of PFTuner is outlined in Algorithm 1. Before the iterative loop begins, an initial observation set *D* is collected, and *m* independent Gaussian Process surrogate models $GP_{1},\ldots ,GP_{m}$ are initialized, one for each optimization objective.

At the start of each iteration, the algorithm first computes the current Pareto front $PF_{t}$ from the accumulated observation set *D* using non-dominated sorting (Line 2). This front serves as the reference baseline against which the potential contribution of any new candidate configuration will be measured in subsequent steps. Next, each pseudo-agent independently maximizes its own acquisition function $AF_{t}\left(\cdot \right)$ to generate a candidate configuration (Line 3). Specifically, each agent $A_{i}$ applies Expected Improvement (EI) over its dedicated surrogate model $GP_{i}$, proposing the configuration that appears most promising from its own single-objective perspective. The union of all *m* proposals forms the candidate set $X_{c}$, which by construction spans diverse trade-off solutions across the objective space.

For each candidate $x\in X_{c}$, the algorithm then enters the MHD improvement evaluation loop (Lines 4–8). The predicted objective values $f^{\mathrm{pre}}$ are rapidly obtained from the corresponding GP surrogate models without triggering any real benchmark execution (Line 5). A hypothetical Pareto front $PF_{t}^{\prime }$ is then constructed by temporarily merging the predicted point into the current front $PF_{t}$ (Line 6), and the MHD-based improvement score I(x) is calculated according to Equation (4) to quantify the marginal geometric gain this candidate would contribute to the overall Pareto front quality (Line 7). After evaluating all candidates, the algorithm selects the single configuration $x_{t+1}$ that yields the maximum improvement score (Line 9). This “propose-and-select” mechanism ensures that every subsequent evaluation is directed toward the direction of greatest Pareto front expansion.

Before committing to a real-world evaluation, the algorithm applies a threshold filtering check (Lines 10–12). If the improvement score $I(x_{t+1})$ exceeds the preset threshold τ=0.005, the configuration is considered sufficiently promising, and a real benchmark evaluation is triggered to obtain the true objective values $f^{\mathrm{act}}\left(x_{t+1}\right)$. Otherwise, the candidate is deemed to offer only marginal gain, and the evaluation is skipped entirely to conserve the limited time budget for more impactful explorations.

If the threshold check is passed, the resulting observation sample $\langle x_{t+1},f^{\mathrm{act}}\left(x_{t+1}\right)\rangle$ is appended to the dataset *D* (Line 13), and all *m* GP surrogate models are retrained on the updated *D* to incorporate the latest empirical evidence (Line 14). The iteration counter is then incremented (Line 15), and the process returns to the next cycle. This loop repeats until the evaluation budget $OT_{\max}$ is exhausted, at which point the non-dominated solutions in *D* constitute the final approximation of the Pareto-optimal configuration set $X^{*}$.
**Algorithm 1:** PFTuner Optimization Process
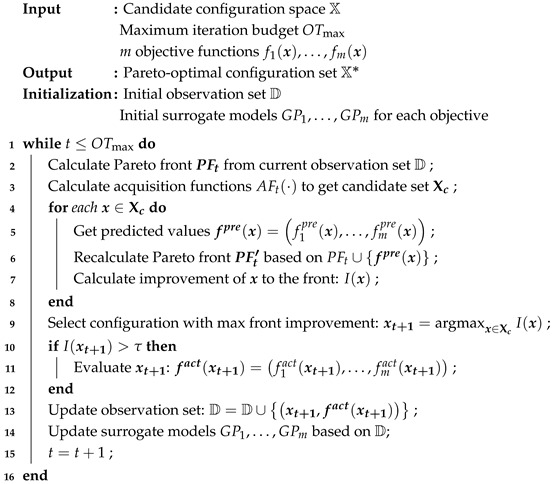


### 4.4. Implementation of PFTuner

This paper implements the main functionalities of PFTuner using Python 3 and utilizes Ansible to achieve automated testing functions. Specifically, this includes the following: using the Sklearn function library to implement the Bayesian Optimization algorithm and the dominance sorting operations for configuration sets; writing Ansible PlayBook scripts to modify the corresponding software configuration files (such as *spark.conf*, *hbase-site.xml*, *my.cnf*, etc.) based on each configuration sample, or issuing system commands to automatically update the new configurations into the respective software systems; simultaneously completing the automated deployment of the cluster servers and launching the corresponding benchmark applications for each software system to conduct stress testing; and using regular expressions to extract the runtime metric values for each software system and persistently storing them in a local database.

## 5. Experimental Setup

### 5.1. Experimental Platform and Workloads

To comprehensively evaluate the tuning performance and cross-system adaptability of PFTuner, we conducted experiments on three widely used software systems, namely Apache Spark, Apache HBase, and MySQL. These systems were deliberately selected to cover diverse application domains and heterogeneous configuration characteristics. Apache Spark is a unified analytics engine for large-scale data processing, and serves as a representative of in-memory computing frameworks with high-dimensional configuration spaces. In contrast, Apache HBase is a distributed NoSQL database designed for scalable storage, where system performance is highly sensitive to I/O-related configurations. MySQL, as a widely used relational database management system, represents traditional transactional workloads, characterized by complex internal mechanisms such as buffer management and locking strategies. By including these fundamentally different systems, the evaluation aims to examine whether PFTuner can generalize effectively across diverse software environments with substantially different performance behaviors and configuration landscapes.

The experiments were conducted on a local cluster composed of three Linux servers, with all nodes connected via a LAN. Each server was equipped with an Intel (R) Core (TM) i7–10700 CPU (16 physical cores), 16GB of DDR4 memory, and a 1TB HDD. The specific hardware and software environment settings are shown in Table 1.

At the same time, to generate workloads for the systems being tuned, three widely used benchmark tools were embedded into the Configuration Evaluator, and their corresponding workloads were selected. Specifically: the PageRank, Aggregation, and Bayes workloads from HiBench were used to benchmark Spark; Workloada and Workloadf from YCSB were used to benchmark HBase; and the ReadOnly, WriteOnly, and ReadWrite workloads from Sysbench were used to benchmark MySQL.

### 5.2. Optimization Objectives and Key Parameter Selection

In this paper, we consider system performance and the corresponding energy consumption as the two primary objectives for configuration tuning across all workloads. Performance-related metrics are obtained from the benchmark tools introduced earlier, while processor energy consumption is measured using the Turbostat tool.

In the context of software configuration optimization, it is essential to identify a subset of influential parameters from the typically large configuration space, which often contains hundreds of candidates. This process significantly reduces the dimensionality of the search space and improves the efficiency of the optimization algorithm. To achieve this, we adopt a systematic multi-stage parameter selection strategy.

We begin with an initial screening phase, where candidate parameters are identified based on official configuration documentation of each software system, including Spark, MySQL, and HBase, as well as insights from prior studies [33,34,35,36,37]. This step yields a reduced set of parameters that are likely to have a substantial impact on key system metrics such as performance and energy consumption. Based on the selected parameter space, we then collect empirical data by performing random sampling. Specifically, for each software system and workload, 200 configuration samples are generated. Each sample consists of a complete configuration and its corresponding runtime metrics, including execution time, throughput, and energy consumption, forming a dataset of 〈configuration, metric set〉 pairs. To quantitatively assess the influence of each parameter, we further employ the Random Forest (RF) model to analyze the collected data. Random Forest is well-suited for capturing complex non-linear relationships and provides interpretable measures of feature importance. For each optimization objective, an independent RF model is trained, enabling us to evaluate the contribution of individual parameters to different objectives. Finally, to account for the multi-objective nature of the problem, we integrate the importance scores across different objectives using a weighted aggregation strategy. This ensures that the selected parameters are not only influential for a single objective but also contribute meaningfully across multiple objectives. As a result, the final parameter set supports balanced optimization between performance and energy consumption.

Through this series of steps, the set of key configuration parameters was ultimately refined and determined. Table 2, Table 3 and Table 4 provide detailed descriptions of the finally selected key configuration parameters for the Spark, MySQL, and HBase software, including their value ranges, default settings, and functional descriptions.

### 5.3. Baseline Methods

To comprehensively evaluate the effectiveness and efficiency of PFTuner, we compared it against the following representative search methods.

- BO (Bayesian Optimization) [38]: Bayesian Optimization is a black-box optimization algorithm known for its high sample efficiency. This method uses a surrogate model (a Gaussian Process in this study) to capture the prior distribution of the objective function. By balancing the trade-off between exploitation and exploration, it generates the next observation point based on an acquisition function (Expected Improvement, EI, in this study). Native Bayesian Optimization only supports a single objective. In this experiment, we will construct separate BO instances for different single objectives (e.g., BO_Time, BO_Energy) to validate the necessity of joint multi-objective optimization.
- RS (Random Search) [39]: Random Search generates configurations for evaluation from the candidate configuration space in a completely random manner during each iteration. This method does not leverage historical information and is prone to getting trapped in local optima during the optimization process. Nonetheless, given the same observation time constraint, this method is often more effective than grid search and is therefore frequently used as a basic performance baseline.
- NSGA-II [40]: Non-dominated Sorting Genetic Algorithm II is an improved, genetic algorithm-based multi-objective search technique used for solving multi-objective optimization problems with expensive black-box functions. Its core lies in using evolutionary processes, such as genetic crossover and mutation, to guide the entire population toward approximating the true Pareto front. It is one of the most classic and widely used algorithms in the field of multi-objective optimization.
- USeMO [41]: USeMO is a recently proposed Uncertainty-aware Multi-objective Search framework. This method generates a Pareto-optimal set of candidate configurations by directly solving a computationally inexpensive multi-objective optimization problem defined by corresponding acquisition functions. It then identifies the single best configuration from this set for real evaluation based on its proposed uncertainty metric. It represents a state-of-the-art approach in the field of multi-objective Bayesian optimization.
- EHVI (Expected HyperVolume Improvement) [42]: Expected HyperVolume Improvement is a classical acquisition function specifically designed for multi-objective Bayesian Optimization. At each iteration, EHVI selects the candidate configuration that maximizes the Expected Improvement in HyperVolume relative to the current Pareto front, thereby simultaneously accounting for both convergence toward the true Pareto front and diversity among the solutions. Compared to scalarization-based approaches, EHVI directly operates in the multi-objective space without requiring any preference weights, making it a principled and widely adopted baseline in the multi-objective Bayesian Optimization literature.

To ensure fairness across all methods, the total number of real-world benchmark evaluations is capped at 50 for every approach. For NSGA-II, whose population-based nature requires explicit control over how evaluations are distributed across generations, we verified through preliminary experiments that the chosen setting of 5 individuals over 10 generations yields the best performance achievable within this budget, ensuring that the inferior results observed for NSGA-II reflect the inherent limitations of population-based methods under strict evaluation constraints rather than suboptimal hyperparameter choices. Similarly, for USeMO and EHVI, we confirmed that the original hyperparameter settings remain appropriate for the problem scales considered in this work.

### 5.4. Evaluation Metrics

We employ two complementary metrics to evaluate the performance of different methods from both qualitative and quantitative perspectives.

First, Pareto front visualization provides an intuitive way to assess multi-objective optimization performance. By plotting the Pareto fronts obtained by different methods in the objective space, the relative quality of solution sets can be directly compared. For minimization problems, a Pareto front that lies closer to the coordinate axes, dominates a larger region, and exhibits a more uniform distribution is generally considered superior. This visualization-based analysis allows for a straightforward comparison of convergence behavior and solution diversity.

In addition to visual inspection, we adopt HyperVolume (HV) [31] as a quantitative metric to measure the overall quality of the obtained Pareto fronts. HV computes the volume (or area in the bi-objective case) of the region dominated by the non-dominated solution set with respect to a predefined reference point, which is typically chosen to be worse than all observed solutions. A larger HV value indicates better performance in terms of both convergence toward the Pareto-optimal front and diversity across the objective space. An important advantage of HV is that it does not require prior knowledge of the true Pareto front, making it particularly suitable for black-box optimization scenarios. In our experiments, all objective values are normalized to the range [0, 1] before computing the HV metric to ensure fair comparisons across different methods.

## 6. Experimental Results

### 6.1. Comparison with Single-Objective Tuning Methods

**Figure 3:**
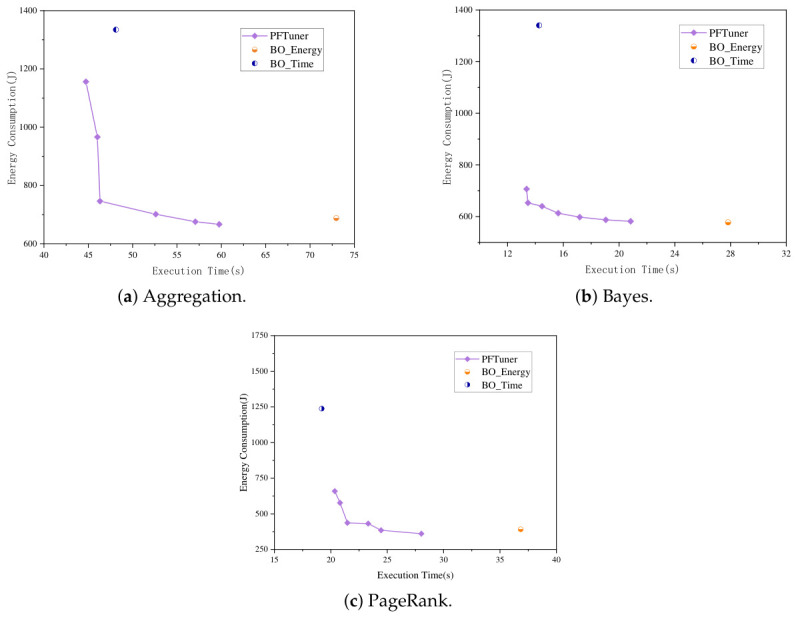
Comparison of Pareto fronts found by PFTuner and single-objective Bayesian Optimization on Spark.

To verify the necessity of joint multi-objective optimization in software configuration tuning, we first take the Spark cluster as an example to evaluate the limitations of traditional single-objective optimization algorithms in multi-objective scenarios. We compared PFTuner with two independent single-objective Bayesian Optimization instances: BO_Time (optimizing only execution time) and BO_Energy (optimizing only energy consumption). As shown in Figure 3, the single-objective baselines (BO_Time/BO_Energy) fall into extreme solutions, failing to address the trade-off nature of the problem. In contrast, PFTuner successfully identifies a balanced Pareto front. This explicitly validates our pseudo-agent strategy in solving conflicting optimization objectives: by employing cooperative agents to represent conflicting interests, the framework ensures that no objective is neglected. The Pareto-optimal set it finds always contains configurations that are close to, or even superior to, the optimal configurations found by the single-objective algorithms.

Specifically, BO_Energy consistently finds a configuration with low energy overhead, but usually at the cost of sacrificing significant execution time. For example, on Spark’s PageRank workload, BO_Energy required 50.64% more task execution time compared to one of the solutions found by PFTuner. In contrast, BO_Time always finds configurations with near-optimal execution time but with significantly higher energy consumption. For example, across all three workloads, the configurations found by PFTuner were able to approach the execution time of BO_Time while simultaneously saving up to 78.84% (Aggregation), 89.69% (Bayes), and 87.74% (PageRank) in energy consumption.

This experimental result clearly demonstrates that in optimization scenarios with multiple conflicting objectives, single-objective optimization algorithms fall into a dilemma of trade-offs, failing to obtain globally balanced, optimal solutions. The fundamental reason lies in the inherent conflict between execution time and energy consumption: configurations that minimize execution time tend to drive up energy consumption, while energy-efficient configurations often come at the cost of longer execution times. A single-objective algorithm can only optimize along one dimension of this trade-off, making the resulting solution suboptimal from a holistic perspective. At the same time, due to the difficulty of pre-defining weights for each objective, simply converting the multi-objective problem into a single-objective one via weighting is also not an effective solution, as any fixed weight assignment risks producing solutions that are poorly suited to the actual operational requirements. In contrast, PFTuner maintains a diverse Pareto-optimal solution set that spans the full trade-off spectrum, allowing operators to select the most appropriate configuration based on their specific deployment context without re-running the optimization.

### 6.2. Comparison with Multi-Objective Tuning Methods

To comprehensively evaluate the effectiveness and efficiency of PFTuner, we compare it with three representative baselines: RS (Random Search), NSGA-II (evolutionary algorithm), and USEMO (uncertainty-aware BO). The comparison focuses on two key aspects: the quality of the discovered Pareto front and the time cost of the optimization process.

#### 6.2.1. Comparison of Pareto Front Quality

**Figure 4:**
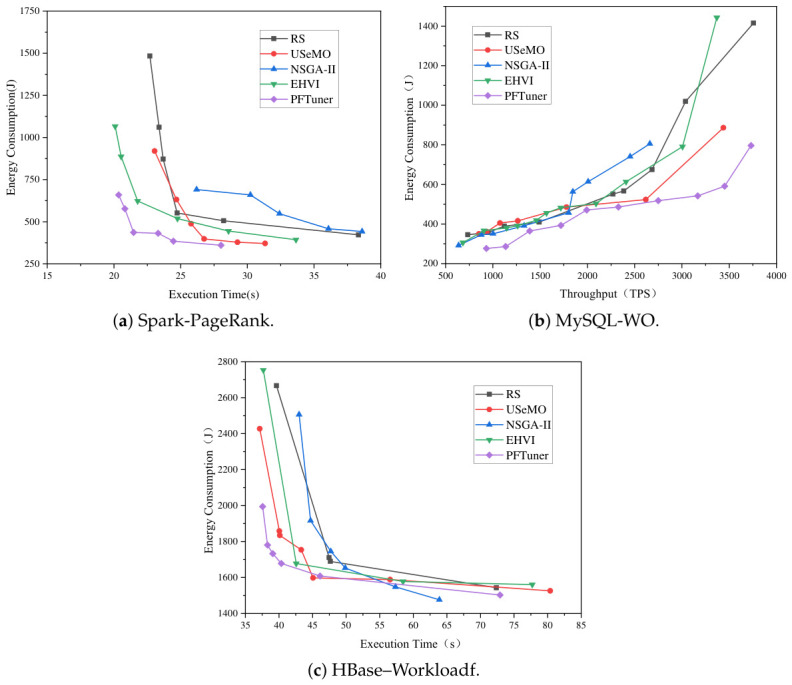
Comparison of Pareto fronts found by PFTuner and baseline methods on three software systems.

Figure 4 illustrates the Pareto fronts identified by PFTuner and the baseline methods across three distinct software systems (Spark, MySQL, HBase) under specific workloads. Visual inspection reveals that PFTuner consistently discovers a superior Pareto front that dominates those of the baselines in most regions.

Specifically, PFTuner achieves a better trade-off between performance and energy consumption compared with the baselines. For the Spark-PageRank workload, compared with the best-performing baseline method, USeMO, PFTuner is able to find a configuration that achieves as much as 10.59% less execution time (28.02 s vs. 31.34 s) with even slightly better minimized energy consumption. In addition, it can also find a configuration that consumes as much as 53.04% less energy (431.82 J vs. 919.62 J) with a fairly close execution time. Compared with EHVI, although EHVI finds a slightly faster minimum execution time (20.09 s vs. 20.35 s), its corresponding energy consumption is 61.77% higher (1066.52 J vs. 659.30 J), indicating that EHVI struggles to balance the two objectives effectively in this scenario. For the HBase–Workloadf workload, to match the execution time achieved by PFTuner, baseline methods require an additional 21.70% to 53.92% energy consumption. Notably, EHVI finds only four Pareto-optimal configurations within the evaluation budget, significantly fewer than PFTuner’s 6, suggesting that its HyperVolume-based acquisition function is less sample-efficient under a strict evaluation constraint. For the MySQL-WO workload, PFTuner achieves the highest throughput on the Pareto front (3730.56 TPS) while maintaining competitive energy consumption and outperforming all baselines, including EHVI, whose Pareto front degrades sharply at the high-throughput end (1442.99 J at 3369.13 TPS vs. 796.7 J at 3730.56 TPS for PFTuner), within the limited evaluation budget.

The consistently superior results of PFTuner across all three platforms can be attributed to two complementary mechanisms. First, the pseudo-agent cooperative strategy decouples conflicting objectives by maintaining independent Gaussian Process surrogate models for each objective, enabling the generation of high-potential candidate configurations $X_{c}$ that are well-distributed across the objective space, rather than being biased toward a single objective as in scalarization-based approaches. Second, the MHD-based selection criterion rigorously quantifies the geometric contribution of each candidate to the current Pareto front without requiring a pre-defined reference point, making it robust to the heterogeneous configuration spaces encountered in different software systems. This "Propose-and-Select" mechanism allows PFTuner to navigate the configuration space more efficiently than NSGA-II, which suffers from poor initialization under strict evaluation budgets, and more robustly than EHVI, whose HyperVolume-based acquisition can be sensitive to the scale and distribution of objective values in irregular spaces.

#### 6.2.2. Optimize Costs Analysis

To explore the search efficiency of different algorithms, we compared the average optimization time overhead per tuning iteration for each method under different Spark workloads. As shown in Table 5, PFTuner achieves the lowest average time overhead across all workloads. Its comprehensive average ptimization time overhead is 24.23 s, 32.49 s, 194.14 s, and 40.78 s lower than RS, NSGA-II, USeMO, and EHVI, respectively.

The time overhead differences among the methods can be attributed to their distinct internal mechanisms. USeMO incurs the highest cost because at each iteration it must solve an internal multi-objective sub-problem using NSGA-II, which involves a full population-based evolutionary process including non-dominated sorting, crowding distance computation, and selection operators. This internal evolutionary process alone constitutes a substantial computational burden that is repeated at every tuning iteration. EHVI’s overhead is the second highest among Bayesian methods. Although EHVI avoids the internal multi-objective sub-problem of USeMO, computing the Expected HyperVolume Improvement acquisition function itself is computationally expensive: its exact calculation requires integrating over the multivariate Gaussian posterior distribution, whose complexity scales super-linearly with both the number of observed points and the number of objectives, making it noticeably slower than simpler acquisition functions such as EI. NSGA-II’s overhead is moderate. While it does not maintain surrogate models, the per-iteration cost still includes population-level genetic operations and non-dominated sorting, which grow with population size. RS exhibits relatively low per-iteration algorithmic overhead since it requires no model fitting or acquisition function optimization. However, because RS has no mechanism to avoid poor configurations, it frequently evaluates configurations with long actual execution times, which inflates its observed average iteration time compared with PFTuner.

PFTuner achieves the lowest overhead through two complementary mechanisms. First, Gaussian Process surrogate models allow PFTuner to recommend high-quality candidate configurations without exhaustive real-world evaluations, and the Expected Improvement acquisition function is computationally cheap to optimize compared with EHVI’s HyperVolume integral. Second, and most critically, the threshold filtering mechanism plays a decisive role: by setting a minimum improvement threshold (τ=0.005), PFTuner intelligently prunes candidate configurations whose predicted MHD gain satisfies I(x)≤τ, thereby avoiding expensive real-world benchmark executions for marginal improvements. This means that PFTuner allocates the limited time budget exclusively to the most promising configurations, effectively decoupling the number of surrogate model queries from the number of costly real-world evaluations. The combined effect of these two mechanisms enables PFTuner to maintain a consistently low per-iteration overhead across all workloads, regardless of the underlying workload characteristics.

### 6.3. Adaptability to Heterogeneous Configuration Spaces

**Figure 5:**
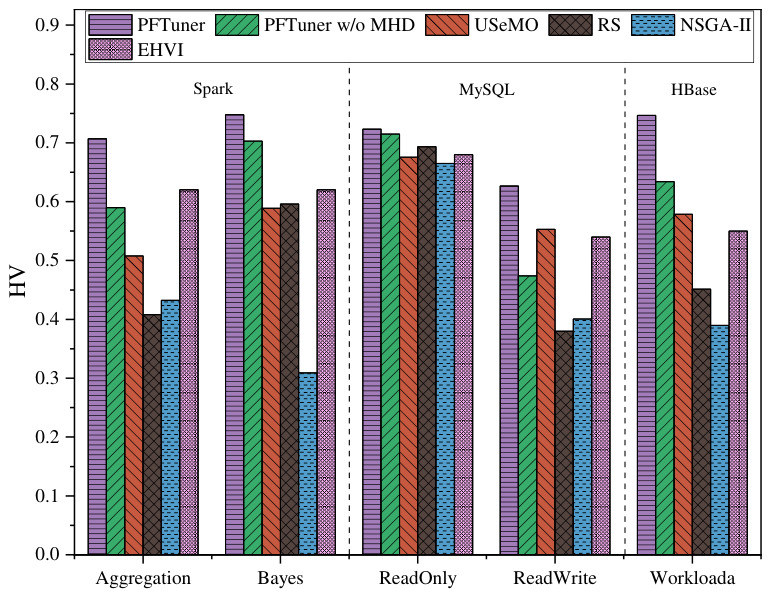
Comparison of HyperVolume (HV) metric values across different workloads.

To evaluate the robustness of PFTuner against heterogeneous configuration spaces, we extended the evaluation to five distinct software-workload scenarios spanning three platforms: Spark (Aggregation and Bayes), MySQL (ReadOnly, ReadWrite), and HBase (Workloada). Figure 5 presents the HyperVolume (HV) metric values for the Pareto fronts obtained by all methods, serving as a quantitative indicator of both convergence and diversity of the solution set. From the results, it is evident that PFTuner consistently achieves the highest HV value across all five scenarios, demonstrating that the comprehensive quality of its solution sets remains superior regardless of the underlying software platform.

Specifically, across the five workloads, PFTuner’s HV metric achieves improvements of 1.04× to 1.73× over RS, 1.07× to 1.39× over USeMO, 1.08× to 2.41× over NSGA-II, 1.06× to 1.36× over EHVI, and 1.01× to 1.32× over PFTuner w/o MHD, respectively. A closer examination reveals that the performance gap between PFTuner and the baselines tends to widen as the configuration space becomes more heterogeneous. On the Spark workloads, where the configuration parameter space is relatively well-structured and uniform, PFTuner already demonstrates clear advantages over all baselines. This advantage becomes even more pronounced on the MySQL ReadWrite and HBase Workloada scenarios, confirming that the MHD-based guidance is increasingly critical as the configuration landscape grows more irregular and sparse. The only exception is the MySQL ReadOnly workload, where the performance differences across all methods are relatively small, consistent with the earlier observation that MySQL’s default configuration already handles read-only workloads efficiently, leaving limited room for further optimization.

The comparison between PFTuner and PFTuner w/o MHD provides direct ablation evidence for the effectiveness of the MHD-based selection criterion. Removing MHD guidance consistently degrades HV performance across all workloads, with the largest drops observed on the more irregular configuration spaces, such as MySQL ReadWrite and HBase Workloada, while the gap becomes much smaller on the relatively uniform MySQL ReadOnly workload. This observation confirms that MHD guidance is particularly beneficial when the objective space geometry is complex, as it helps prevent the search process from being trapped in low-quality regions by providing a stable geometric optimization direction. Furthermore, PFTuner consistently outperforms EHVI across all workloads. Although EHVI also optimizes directly in the multi-objective space, its computational complexity increases rapidly with the number of observations, limiting its search efficiency under a fixed budget. In contrast, the MHD metric offers a similarly principled geometric criterion with significantly lower computational overhead, enabling PFTuner to allocate more evaluations to promising configurations. More importantly, unlike previous heuristic-based methods that implicitly assume relatively uniform parameter spaces, PFTuner decouples the search strategy from system-specific characteristics through a purely geometric optimization objective, thereby maintaining strong adaptability across heterogeneous software platforms without requiring domain-specific adjustments.

### 6.4. Case Study of Representative Configurations

**Figure 6:**
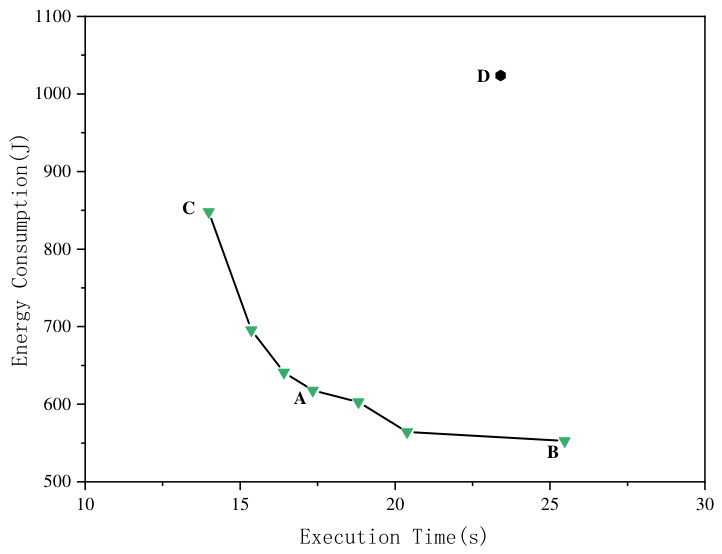
Representative configuration points on Spark–Bayes workload.

To gain a deeper understanding of the role multi-objective optimization plays in specific parameter selection, we selected four representative configuration points (A, B, C, D) from PFTuner’s search process on the Spark–Bayes workload for analysis, as shown in Figure 6.

- Point A represents the best-balanced configuration, achieving dual optimization of both performance and energy consumption.
- Point B is the configuration with the lowest energy consumption among all Pareto solutions, suitable for scenarios with extreme requirements for energy control.
- Point C achieves the shortest task execution time, suitable for scenarios with extreme performance demands.
- Point D is a dominated solution, performing poorly in both performance and energy consumption, representing a case of configuration imbalance.

Table 6 summarizes some of the key parameter values for these four configuration points. It can be clearly seen that different configuration values directly impact the system’s performance and energy consumption. For example, to achieve extreme performance, Point C adjusted the CPU frequency (userspace_frequency) to 3.50 GHz and set spark.memory.fraction to 0.9, dedicating the majority of executor memory to caching and shuffle operations; users with latency-sensitive requirements are recommended to pay attention to this configuration. Point B, aiming for energy savings, reduced userspace_frequency to 1.70 GHz while allocating more memory to spark.executor.memory (4096 MB) to compensate for the reduced CPU throughput; users whose primary concern is energy efficiency are recommended to pay attention to this configuration. Point A operates at a moderate CPU frequency (2.90 GHz) with a conservative spark.memory.fraction of 0.4, delivering a balanced trade-off between execution time and energy consumption; for most production deployments, users are recommended to pay attention to this configuration as a practical starting point. Point D serves as a cautionary example: despite its seemingly reasonable mid-range parameter settings (userspace_frequency = 2.00 GHz, spark.memory.fraction = 0.7), it yields poor results in both objectives simultaneously due to suboptimal interactions among parameters. This highlights the necessity of adopting a joint multi-objective tuning method. Only through meticulous balancing and adjustment of various parameters can optimal trade-offs between different objectives be found.

## 7. Conclusions

In this paper, we propose PFTuner, an efficient and effective multi-objective configuration tuning framework adaptive to different software systems. PFTuner consists of three cooperating modules: Configuration Generator, Configuration Evaluator, and Sample Collector. At its core, the Configuration Generator employs a pseudo-agent cooperative strategy to decouple conflicting optimization objectives into independent Gaussian Process surrogate models and incorporates the Modified HyperVolume-based Difference (MHD) metric to guide the search toward configurations that maximally expand the Pareto front. Beyond candidate generation, a threshold filtering step gates real benchmark executions behind a minimum MHD improvement score, so the tight evaluation budget is spent only where it matters and not squandered on runs whose predicted gain is negligible. Extensive experiments across eight different software-workload scenarios on three widely used software systems demonstrate that PFTuner consistently outperforms representative baseline methods in Pareto front quality, search efficiency, and cross-system adaptability. Compared with the best-performing baseline, PFTuner achieves significant improvements in both execution time and energy consumption while reducing the average optimization time overhead per iteration by up to 194.14 s. Furthermore, PFTuner achieves HV metric improvements of 1.04×–2.41× over all baselines across heterogeneous software-workload scenarios, demonstrating robust adaptability regardless of the underlying configuration space structure. In the future, we intend to extend PFTuner to support more software systems and to investigate whether transfer learning and meta-learning can shorten the cold-start phase when moving to a previously unseen deployment environment.

## Figures and Tables

**Table 1:** Experimental hardware and software environment.

	CPU	Intel (R) Core (TM) i7–10700
Hardware Environment	RAM	DDR4 16 GB
	Storage	1 TB HDD
	OS	Ubuntu 20.04.3LTS
	Hadoop	2.7.3
	Spark	2.2.2
	MySQL	5.7.32
Software Environment	HBase	2.0.5
	HiBench	7.0
	Sysbench	1.0.18
	YCSB	0.17.0
	Turbostat	19.08.31

**Table 2:** The description of Spark configuration parameters.

Parameter Name	Default Value	Value Range	Description
spark.driver.memory	1024	1024–4028	Max heap size for driver
spark.executor.memory	1024	1024–4028	Max heap size for executor
spark.executor.core	3	1–4	Number of cores for executor tasks
spark.shuffle.sort.bypassMergeThreshold	200	100–1000	Threshold for bypassing merge-sort phase
spark.shuffle.accurateBlockThreshold	100 × $1024^{2}$	[100–1000] × $1024^{2}$	Threshold for recording accurate partitions
spark.broadcast.blockSize	4	2–128	Block size for broadcast variables
spark.default.parallelism	32	8–128	Default parallelism for Spark jobs
spark.io.compression.lz4(snappy).blockSize	32	2–128	Block size for data compression
spark.memory.fraction	0.6	0.3–0.9	Fraction of executor memory for storage/cache
userspace_frequency	1.7 GHz	0.8–3.8 GHz	CPU Frequency
-XX:+“GC”	Parallel	Serial, Parallel, CMS, G1	Garbage Collector
vm.dirty_ratio	20	10–100	Max percentage of dirty pages in memory
vm.swappiness	60	0–100	Degree of system memory usage by swapper
block.nr_requests	128	64–256	Size of the block device request queue

**Table 3:** The description of MySQL configuration parameters.

Parameter Name	Default Value	Value Range	Meaning
innodb_buffer_pool_instances	8	1–64	Number of InnoDB buffer pool instances
innodb_buffer_pool_size	1	1–8	Size of the InnoDB buffer pool
innodb_doublewrite	on	on/off	Whether to enable the doublewrite buffer
innodb_flush_log_at_trx_commit	1	0/1/2	Controls when the transaction log is written and flushed to disk
innodb_log_files_in_group	2	2–20	Number of files in the redo log file group
innodb_purge_threads	4	1–32	Number of threads for purge operations
innodb_read_ahead_threshold	56	0–64	Threshold to trigger a read-ahead operation
innodb_thread_concurrency	0	0–128	Number of concurrent threads within the InnoDB engine
join_buffer_size	2	1–128	Join buffer size
max_allowed_packet	4	1–512	Maximum allowed size of a single packet during communication
max_heap_table_size	16	4–1024	Maximum size of memory temporary tables
query_prealloc_size	8	8–1024	Fixed buffer size for query parsing
sort_buffer_size	2	1–128	Memory buffer size for sort operations
thread_cache_size	−1	−1–1024	Thread cache size
userspace_frequency	1.7 GHz	0.8–3.8 GHz	CPU Frequency

**Table 4:** The description of HBase configuration parameters.

Parameter Name	Default Value	Value Range	Description
hbase.client.max.perregion.tasks	1	1–5	Max tasks for HRegion data update
hbase.client.max.perserver.tasks	2	2–20	Max tasks for HRegion Server data update
hbase.hregion.memstore.flush.size	8 × 16 MB	[4–32] × 16 MB	MemStore memory limit size
hbase.hstore.compactionThreshold	3	3–6	Threshold for HFile to trigger Compaction
hbase.ipc.server.callqueue.read.ratio	0.0	0.0–1.0	Ratio of read operation queue
hbase.ipc.server.callqueue.scan.ratio	0.0	0.0–1.0	Ratio of scan operation queue
hbase.regionserver.global.memstore.upperLimit	0.4	0.1–0.6	Upper limit of memory ratio occupied by MemStore data
hbase.regionserver.handler.count	30	20–80	Number of RPC listener instances on HRegion Server
hbase.rs.cacheblocksonwrite	false	true/false	Whether to cache data blocks after persistence
hbase.storescanner.parallel.seek.enable	false	true/false	Whether to enable parallel data scanning
hbase.heapsize	1 G	[1–16] G	HBase heap memory size
hfile.block.bloom.cacheonwrite	false	true/false	Whether Bloom filter is cached to BlockCache
io.storefile.bloom.block.size	2 × 64 KB	[1–10] × 64 KB	Data block size in Bloom filter
userspace_frequency	1.7 GHz	0.8–3.8 GHz	CPU Frequency

**Table 5:** Average optimization time overhead per tuning iteration under different Spark workloads.

Algorithm	PFTuner	RS	NSGA-II	EHVI	USeMO
Workload					
Aggregation	**133.81 s**	150.20 s	173.28 s	172.43 s	316.47 s
Bayes	**92.43 s**	131.23 s	122.04 s	143.15 s	289.38 s
PageRank	**107.03 s**	124.53 s	135.43 s	140.03 s	309.86 s

**Table 6:** Key parameter values of representative configuration points on Spark–Bayes workload.

Parameter Name	A	B	C	D
spark.driver.memory	4096	1024	1024	2048
spark.executor.memory	1024	4096	4096	2048
spark.broadcast.blockSize	8	4	16	4
spark.memory.fraction	0.4	0.5	0.9	0.7
userspace_frequency	2.90 GHz	1.70 GHz	3.50 GHz	2.00 GHz
block.nr_requests	70	64	89	87

## Data Availability

Data will be made available on request.
